# Cardiovascular magnetic resonance in contemporary guidelines: divergence between ESC and NICE across major cardiovascular diseases

**DOI:** 10.1093/ehjimp/qyag079

**Published:** 2026-04-22

**Authors:** Nicholas Sawh, Rui Li, Gareth Matthews, Andrew J Swift, Amardeep Dastidar, David P Ripley, Peter P Swoboda, Pankaj Garg

**Affiliations:** Department of Cardiovascular and Metabolic Health, Norwich Medical School, University of East Anglia, Research Park, Norwich NR4 7TJ, UK; Department of Cardiovascular and Metabolic Health, Norwich Medical School, University of East Anglia, Research Park, Norwich NR4 7TJ, UK; Department of Cardiology, Norfolk and Norwich University Hospitals NHS Foundation Trust, Norwich Research Park, Norwich NR4 7UY, UK; Department of Cardiovascular and Metabolic Health, Norwich Medical School, University of East Anglia, Research Park, Norwich NR4 7TJ, UK; Department of Cardiology, Norfolk and Norwich University Hospitals NHS Foundation Trust, Norwich Research Park, Norwich NR4 7UY, UK; Division of Clinical Medicine, Immunity & Cardiovascular Disease, University of Sheffield, Sheffield S10 2RX, UK; Sheffield Biomedical Research Centre, National Institute for Health and Care Research, Sheffield S1 4DA, UK; Department of Cardiology, North Bristol NHS Trust, Bristol BS10 5NB, UK; Department of Cardiology, Bristol Heart Institute, Bristol BS2 8ED, UK; Department of Cardiology, Northumbria Healthcare NHS Foundation Trust, Newcastle NE27 0QJ, UK; School of Medicine, University of Sunderland, Sunderland SR1 3SD, UK; Leeds Institute of Cardiovascular and Metabolic Medicine, University of Leeds, Leeds LS2 9JT, UK; Department of Cardiovascular and Metabolic Health, Norwich Medical School, University of East Anglia, Research Park, Norwich NR4 7TJ, UK; Department of Cardiology, Norfolk and Norwich University Hospitals NHS Foundation Trust, Norwich Research Park, Norwich NR4 7UY, UK

**Keywords:** cardiovascular magnetic resonance, European Society of Cardiology, National Institute for Health and Care Excellence, clinical practice guidelines, diagnostic imaging, health policy

## Abstract

Cardiovascular magnetic resonance (CMR) has transitioned from a specialist tool to a pivotal, multiparametric modality shaping diagnosis, risk, and therapy across major cardiovascular diseases; however, guideline positioning remains inconsistent between the European Society of Cardiology (ESC) and the National Institute for Health and Care Excellence (NICE). We performed a structured synthesis of contemporary ESC and NICE guidance (2015–25), mapping recommendation strength, evidential certainty, and pathway sequencing across heart failure, cardiomyopathies, ischaemic, and valvular disease, anchored to a curated PubMed dataset for transparency and reproducibility. Thirteen ESC and five NICE documents met the inclusion. This was a structured guideline review with narrative policy synthesis. ESC integrates CMR early with graded, disease-specific roles for morphology, tissue characterization, stress perfusion, and flow quantification; NICE adopts a more conservative, problem-solving stance, typically after echocardiography or CT triage. Concordance is strongest in heart failure and valvular disease, whereas divergence persists in cardiomyopathies and myocarditis, and in chronic coronary syndromes, where CT-first pathways dominate in NICE. These differences alter diagnostic yield, time-to-aetiology, radiation exposure, and downstream allocation to revascularization, electrophysiology, genetic testing, and surveillance, with measurable implications for equity within capacity-constrained services. Methodological drivers include ESC’s emphasis on clinical utility once analytic validity is established vs. NICE’s explicit cost-effectiveness modelling and deliverability thresholds. Aligning recommendations around shared outcomes, rapid-update pathways, and commissioning levers would reduce unwarranted variation while preserving fiscal responsibility. This review defines where and why CMR’s value is realized or deferred in UK practice, and proposes pragmatic, evidence-linked strategies to harmonize guidance and accelerate mechanism-based care across populations, regions, and clinical acuity levels.

## Graphical Abstract

**Graphical Abstract:**
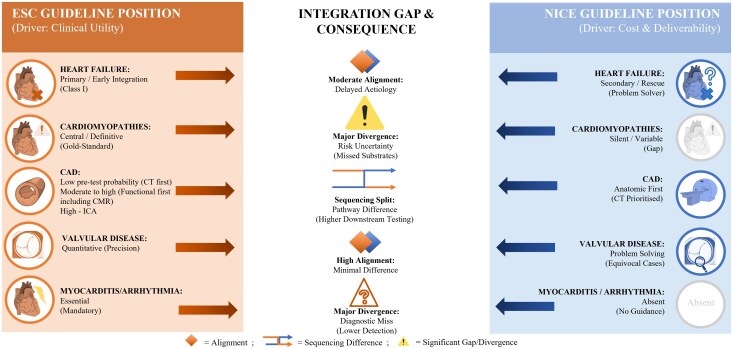
Role of CMR in ESC guidelines vs. NICE guidelines. ESC, European Society of Cardiology; NICE, National Institute for Health and Care Excellence.

## Introduction

Cardiovascular disease remains the leading global cause of morbidity and mortality, and ageing populations and multimorbidity continue to amplify lifetime risk. Imaging is central to contemporary cardiovascular care because it can quantify subclinical disease, refine risk stratification, and track therapeutic response across diverse phenotypes. Beyond echocardiography, cross-sectional modalities characterize coronary, myocardial, and valvular pathology with reproducible metrics that can align research with practice. Coronary calcium scoring, CT angiography, and cardiac magnetic resonance (CMR) can improve reclassification beyond traditional scores and may reveal sex- and ethnicity-specific patterns, thereby supporting more precise prevention. Integrating imaging into prevention pathways therefore shifts practice from late symptomatic detection towards earlier, mechanism-based intervention.^1^

CMR is now used routinely rather than only in specialist centres to guide diagnosis, aetiology, and risk. It uniquely combines high-fidelity volumetrics, tissue characterization, perfusion, and flow quantification without ionizing radiation, allowing one examination to answer multiple clinical questions. Late gadolinium enhancement and mapping stratify arrhythmic and heart failure risk across ischaemic and non-ischaemic substrates, guide device selection, and inform therapy response. Stress perfusion CMR rivals invasive physiology for ischaemia assessment, while right ventricular and pulmonary haemodynamic surrogates transform pulmonary hypertension care. Consequently, ESC guidelines increasingly position CMR across diagnostic and prognostic pathways in heart failure and cardiomyopathies, within contemporary European practice.^2^

Clinical guidelines convert evidence into standardized care; however, scopes and levers differ between the European Society of Cardiology (ESC) and the National Institute for Health and Care Excellence (NICE). ESC guidelines synthesize pan-European evidence and grade recommendations to optimize outcomes across healthcare settings, often integrating emerging technologies once analytic validity and clinical utility are persuasive. NICE, by contrast, embeds cost-effectiveness modelling, commissioning implications, and UK service deliverability, sometimes prioritizing budget impact and implementation feasibility. In heart failure, for example, CMR is integrated by ESC for aetiology and risk stratification, whereas NICE adoption has been more selective and pathway-specific to date.^1–3^

For UK cardiologists, such divergence can complicate operational decision-making. ESC pathways may encourage earlier CMR or stress imaging when diagnostic yield and prognostic value are strong. NICE may sequence tests differently to reflect cost-effectiveness or capacity constraints, producing parallel but non-identical routes to angiography or revascularization. Studies comparing ESC and NICE diagnostic strategies report similar invasive angiography yields, but different upfront imaging choices and costs, which can create uncertainty in resource-limited services.^4,5^ This review addresses those tensions using a structured framework mapped to disease domains and UK commissioning realities, underpinned by a curated PubMed dataset described in the Methods section, to support transparency and reproducibility.

This review aims to compare how CMR is positioned across ESC and NICE guidance, describe sources of divergence, and examine clinical, economic, and equity consequences for UK practice. Scope includes heart failure, cardiomyopathies, ischaemic and valvular disease, with pragmatic proposals for alignment and implementation across commissioning pathways within the NHS.

Beyond descriptive comparison, we provide a pragmatic interpretive appraisal. This section should be read as a synthesis of the cited evidence and guideline positions, supplemented by expert interpretation where direct comparative data are limited. We outline where earlier CMR adoption is most likely to improve diagnostic confidence or risk stratification, where conservative sequencing may remain justified by pre-test probability and deliverability, and where hybrid pathways may better align evidential value with system constraints.

## Methods

### Study design

#### Design and reporting framework

**Figure 1:**
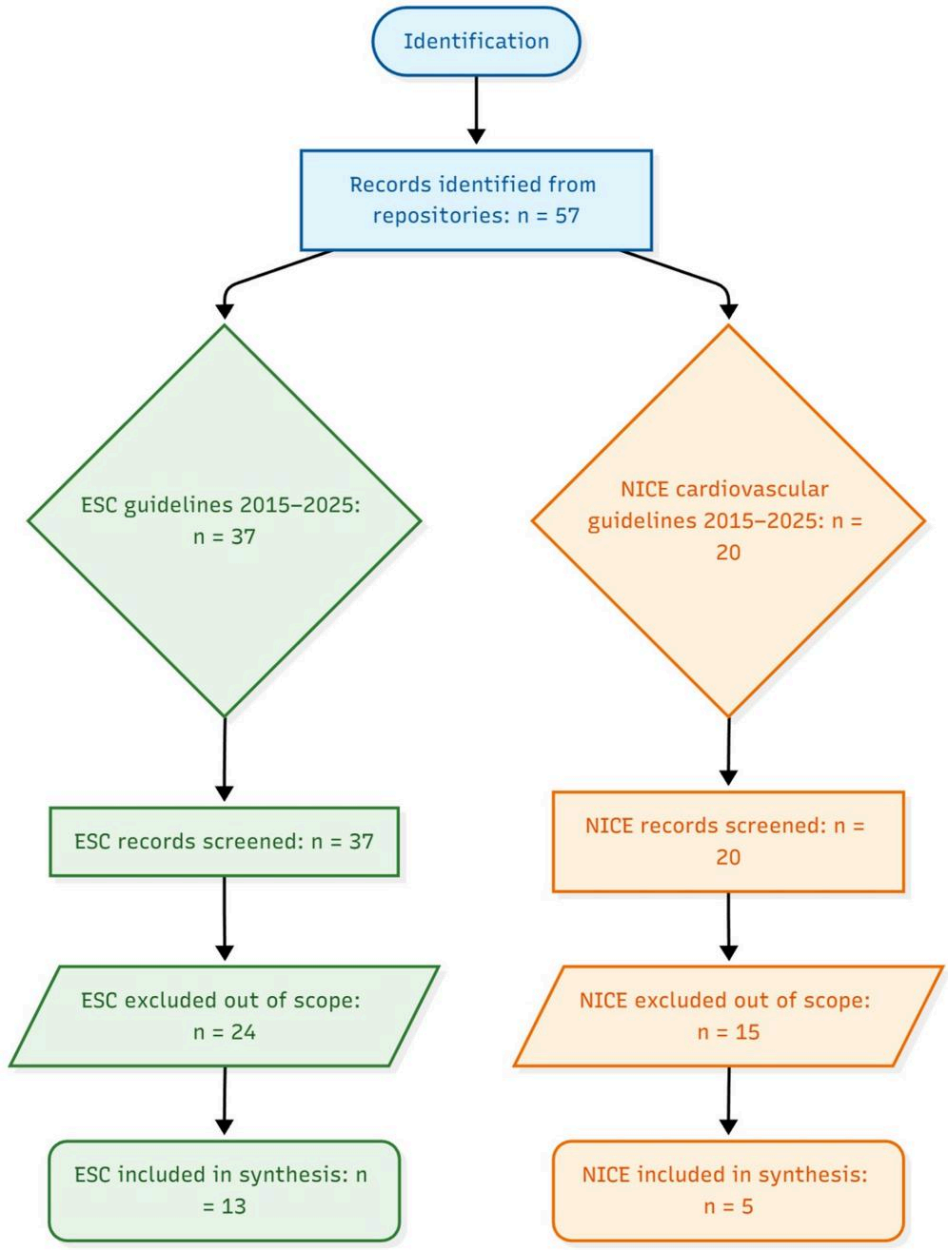
European Society of Cardiology; NICE, National Institute for Health and Care Excellence.

This work is best interpreted as a structured guideline review with narrative policy synthesis (systematic search and selection of guidance documents followed by domain-based comparative synthesis), rather than a systematic review of primary clinical studies or a scoping review. PRISMA 2020 was used to report the search, screening, and inclusion process (*Figure 1*). AGREE II–informed qualitative appraisal was used to contextualize methodological quality of included guidance documents; we did not compute item-by-item AGREE II scores, but summarized domain-level strengths and limitations for each included document (see Supplementary data online, *Table S2*). GRADE concepts were used as an interpretive lens to contextualize evidential certainty and explain how differing organizational frameworks (ESC class/level vs. NICE narrative directives) can yield different recommendation strength; we did not re-grade the underlying evidence beyond the grading already reported by the guideline bodies. CHEERS 2022 was used as a conceptual benchmark to structure the extraction and reporting of economic considerations referenced within NICE guidance and the supporting literature; it was not used as a checklist-based appraisal of each individual economic model. For this review, the curated PubMed dataset denotes the reviewer-assembled set of PubMed-indexed records linked to included guidelines, methodology papers, and key disease-domain evidence during screening and extraction. It served as an auditable reference set rather than as a stand-alone dataset for quantitative synthesis.

Guideline identification strategy targeted the ESC and NICE repositories, supplemented by PubMed to link each guideline to any companion methodology or evidence summary. Two reviewers independently screened titles and versions, including adult-focused guidelines and technology appraisals with explicit diagnostic recommendations. We excluded paediatric-only documents, position statements lacking formal grading, and pathway tools without evidence-based rationale. Data extraction captured development methods, grading systems, and all statements positioning CMR relative to alternatives. Reporting followed a PRISMA-style flow adapted for guideline synthesis, and methodological quality was contextualized with AGREE II and Institute of Medicine trustworthiness standards to interpret divergences arising from process rather than evidence^6–8^ (*Figure 1*).

### Ethics approval and consent to participate

Not applicable for this review of published guidelines and consensus statements.

### Settings and participants

Disease domains and timeframe were specified *a priori* to maximize generalizability and policy relevance. We focused on heart failure, cardiomyopathies, ischaemic heart disease (acute and chronic), and valvular heart disease—conditions with high burden and mature imaging pathways in which CMR plausibly alters diagnosis, risk assessment, or treatment allocation. The sampling window encompassed the latest major ESC and NICE versions and updates in the last decade, while retaining earlier anchor documents where still active or explicitly cross-referenced.

### Outcomes

For each disease domain and time point, data extraction captured development methods, grading systems, and all statements positioning CMR relative to alternative modalities. Recorded parameters included recommendation class or strength, level or certainty of evidence, and any economic, capacity, or commissioning qualifiers influencing implementation. Comparative analysis aligned ESC classes and evidence levels with the narrative or directive format used by NICE, facilitating interpretation of differences in clinical positioning, timing, and utilization thresholds.

### Methodological appraisal and interpretive framework

As outlined above, GRADE and CHEERS were used as interpretive benchmarks (not formal re-appraisals), and AGREE II–informed qualitative appraisal was applied (see Supplementary data online, *Table S2*). No formal statistical pooling was undertaken. Instead, evidence-based certainty was interpreted through GRADE domains to understand how comparable datasets led to differing recommendations. Diagnostic sequences and substitution rules were mapped to identify concordance, upgrading, or downgrading across iterations. Economic arguments, commissioning caveats, and capacity assumptions were abstracted and benchmarked against CHEERS 2022 to distinguish evidence statements from cost-effectiveness focus. AGREE II–informed qualitative appraisal was used to contextualize divergences when conflicts persisted, ensuring that apparent differences were judged in the light of transparency, stakeholder engagement, and implementability.^9–12^

## Results

### Primary outcomes

From 2015 to 2025, 57 guideline records were identified across ESC repositories and NICE cardiovascular guidance. Thirty-seven ESC documents and 20 NICE documents underwent screening. Twenty-four ESC and 15 NICE records were excluded as out of scope, yielding 13 ESC and 5 NICE guidelines included in the synthesis. The PRISMA pathway is summarized in the flow diagram and underpins comparative analyses of CMR positioning across disease domains (*Figure 1*). This corpus provides a balanced sample of contemporary European and UK guidance with sufficient breadth to interrogate concordance, divergence, and evidential grading for CMR indications, while limiting heterogeneity by excluding documents outside prespecified cardiovascular scope (*Figure 1*).

The included ESC guidelines comprised cardiomyopathies,^13^ heart failure,^2^ myocarditis and pericarditis,^14^ chronic coronary syndromes,^15^ non-ST-segment elevation acute coronary syndromes,^16^ ST-segment elevation myocardial infarction,^17^ valvular heart disease,^18^ adult congenital heart disease,^19^ ventricular arrhythmias and sudden cardiac death,^20^ atrial fibrillation,^21^ pericardial diseases,^22^ pulmonary hypertension,^23^ and infective endocarditis^24^ (*Table 1*). The NICE comprised chronic heart failure,^25^ acute heart failure,^26^ chest pain,^27^ acute coronary syndromes,^28^ and heart valve disease^29^ (*Table 2*).

**Table 1:** ESC guidelines with CMR content (2015–25)

Domain	ESC guideline	Year	Citation
Cardiomyopathies	Management of cardiomyopathies	2023	^13^
Heart failure	Acute and chronic HF	2021	^2^
Myocarditis and pericarditis	Management of myocarditis and pericarditis	2025	^14^
Chronic coronary syndromes	Chronic coronary syndromes	2019	^15^
NSTE-ACS	Non-ST-segment elevation ACS	2020	^16^
STEMI	ST-segment elevation MI	2017	^17^
Valvular heart disease	Valvular heart disease (ESC/EACTS)	2021	^18^
Adult congenital heart disease	Adult congenital heart disease	2020	^19^
Ventricular arrhythmias and SCD	Ventricular arrhythmias and SCD	2022	^20^
Atrial fibrillation	Atrial fibrillation	2020	^21^
Pericardial disease	Pericardial diseases	2015	^22^
Pulmonary hypertension	Pulmonary hypertension (ESC/ERS)	2022	^23^
Infective endocarditis	Infective endocarditis	2023	^24^

ESC, European Society of Cardiology; HF, heart failure; NSTE-ACS, non-ST-elevation acute coronary syndromes; STEMI, ST-elevation myocardial infarction; SCD, sudden cardiac death.

**Table 2:** NICE guidelines in scope (2010–25)

Topic	NICE ID	Year (last update)	Status
Chronic heart failure in adults	NG106	2018 (updated 2025)	Active
Acute heart failure	CG187	2014 (updated 2021)	Active
Chest pain of recent onset	CG95	2010 (updated 2016)	Active
Acute coronary syndromes	NG185	2020	Active
Heart valve disease in adults	NG208	2021	Active

NICE, National Institute for Health and Care Excellence.

### Secondary/exploratory outcomes

Across the 18 included guidelines, CMR was positioned variably but with consistent clinical weight in heart failure and valvular disease, where ESC affords graded, class-labelled recommendations mirrored by NICE when echocardiography is inadequate^2,18^ (*Table 3*). Broader deployment appears in ESC cardiomyopathy and myocarditis pathways, emphasizing tissue characterization, fibrosis burden, and risk stratification, areas where NICE offers little explicit direction.^13,14^ In ischaemic pathways, ESC privileges stress CMR and viability assessment, whereas NICE often sequences CT first, generating partial concordance and pathway divergence^15,16^ (*Table 3*).

**Table 3:** Cross-comparison of CMR recommendations: ESC vs. NICE (2015–25)

Disease domain	ESC guidance (year; positioning)	NICE guidance (year; positioning)	Concordance	Indicative clinical/system implications
HF	HF 2021: CMR when echo inadequate/inconclusive for LV/RV structure and function (Class I, LoE B); tissue characterization/differential diagnosis	NG106 (2018; updated 2025): MRI suggested if echo inadequate or diagnostic uncertainty persists (no formal grading)	Broad alignment; ESC earlier mechanism-finding, NICE typically later	Potentially earlier aetiology and risk stratification; may reduce repeat testing and speed downstream therapy decisions where indicated
CM	Cardiomyopathies 2023: CMR central for diagnosis, fibrosis, risk stratification (Class I, LoE A); reduces need for biopsy in many settings	No cardiomyopathy-specific imaging guideline (often cross-ref HF NG106)	ESC comprehensive; NICE gap	May support earlier phenotype definition and family screening; could improve SCD risk stratification, reduce invasive biopsy use, and standardize surveillance
IHD	CCS 2019; NSTE-ACS 2020; STEMI 2017: Pre-test probability directed approach. Stress CMR for diagnosis/viability (Class I–IIa, LoE A–B)	CG95 (2016); NG185 (2020): CMR may be considered when CT/echo inconclusive; CT often first-line	Partial concordance; ESC higher evidence weighting for functional CMR; NICE more CT-first	CT is well suited to ruling out coronary artery disease in lower-risk populations. Stress CMR additionally provides ischaemia, function, and scar/fibrosis assessment, while repeat CT-based testing may increase cumulative radiation exposure
VHD	ESC/EACTS 2021: CMR for regurgitant volume and ventricular quantification (Class I, LoE B)	NG208 (2021): Consider MRI when echo inadequate	Strong concordance	More reproducible quantification when echo is discordant; could influence intervention timing and follow-up intervals
ACHD/PH	ACHD 2020; PH 2022: routine CMR for anatomy and RV assessment (Class I, LoE B)	No stand-alone imaging directive (covered variably in TAs)	ESC strong; NICE gap	Potentially improved RV and anatomic assessment; supports longitudinal follow-up and may reduce cumulative radiation from repeated CT surveillance
Inflammatory/infective	Myocarditis/Pericarditis 2025; Pericardial 2015; Endocarditis 2023: CMR for oedema, fibrosis, differential diagnosis (Class I, LoE B)	No explicit NICE imaging guidance	Partial alignment (ESC detailed; NICE largely absent)	May improve diagnostic certainty and prognostic stratification (activity/scar); could alter activity restriction, follow-up intensity, and further testing
Rhythm disorders	AF 2020; VA/SCD 2022: CMR for scar/substrate characterization (Class IIa–IIb, LoE B/C)	No explicit NICE imaging guidance	ESC integrated; NICE omission	May refine arrhythmic risk and procedural planning; could influence ICD selection and ablation strategy beyond EF-only criteria

CMR, cardiovascular magnetic resonance; ESC, European Society of Cardiology; HF, heart failure; LV, left ventricle; RV, right ventricle; MRI, magnetic resonance imaging; CCS, chronic coronary syndrome; NICE, National Institute for Health and Care Excellence; NSTE-ACS, non-ST-segment elevation acute coronary syndrome; STEMI, ST-segment elevation myocardial infarction; CT, computed tomography; ACHD, adult congenital heart disease; PH, pulmonary hypertension; TA, technology appraisal; AF, atrial fibrillation; VA, ventricular arrhythmia; VHD, valvular heart disease; SCD, sudden cardiac death.

## Discussion

This narrative review identified systematic differences between the NICE and ESC frameworks for CMR use. NICE generally positions CMR as a problem-solver after echocardiography or CT triage, whereas ESC embeds CMR earlier within disease-specific, graded recommendations. Concordance is strongest in heart failure and valvular disease. Divergence persists in chronic coronary syndromes and myocarditis, reflecting distinct evidential hierarchies and service assumptions across settings.^2,14,15,18^

### Overview of ESC recommendation

ESC guideline methodology assigns a class of recommendation (I, IIa, IIb, and III) alongside levels of evidence (A, B, and C), and within this framework, cardiovascular magnetic resonance is embedded as a disease-specific, graded intervention rather than a last-line problem solver.^2^ In heart failure, CMR is Class I for defining ventricular morphology when echocardiography is suboptimal and for tissue characterization to resolve aetiology, typically Level B.^2^ In cardiomyopathies, CMR is positioned as central to diagnosis, phenotyping, and risk stratification, with Class I, Level A recommendations where late gadolinium enhancement and mapping alter management.^13^ In ischaemic pathways, stress perfusion CMR and viability imaging occupy Class I to IIa positions for diagnosing flow-limiting disease and adjudicating revascularization candidacy in chronic and post-ACS settings.^15–17^ For valvular disease, CMR is Class I when accurate quantification of regurgitant volumes, right ventricular function, or aortic dimensions is required, particularly when transthoracic windows are inadequate.^18^ The updated myocarditis and pericarditis guidance elevates oedema and scar imaging as Class I tools for diagnosis and activity assessment, integrating mapping with late enhancement to standardize case definition.^14^ Thus, ESC places CMR early to improve diagnosis, risk, and therapy meaningfully.

CMR changes decisions rather than merely describing anatomy. Diagnostic yield derives from multiparametric integration: cine volumes define remodelling and right ventricular involvement; T1/T2 mapping and late gadolinium enhancement separate infarction, myocarditis, infiltrative disorders, and tachycardiomyopathy, resolving causes of heart failure and troponin-positive presentations where echocardiography is indeterminate.^2,14^ Prognostically, scar pattern and burden refine risk across substrates; mid-wall and diffuse fibrosis in dilated and hypertrophic phenotypes track arrhythmic events and pump failure beyond ejection fraction, informing surveillance and the threshold for device therapy.^13,20^ Therapeutically, stress perfusion quantifies ischaemic burden and guides revascularization vs. optimized medical therapy, while viability assessment identifies hibernation and adverse remodelling trajectories after acute coronary syndromes.^15,16^ In valvular disease, phase contrast and 3D volumetry refine regurgitant severity and ventricular response, anchoring timing of intervention when echocardiographic datasets are discordant or limited.^18^ The unifying principle is that a single non-ionizing examination links cause, prognosis, and treatment choice, reducing diagnostic delay and unnecessary invasive testing.^2^

Large registries and multicentre pragmatic studies consistently demonstrate that CMR reclassifies diagnoses, provides incremental prognostic information, and alters management across common referral indications. In undifferentiated heart failure and cardiomyopathy clinics, incorporation of mapping and scar imaging resolves aetiology in a substantial minority, with downstream modification of pharmacotherapy, family screening, and device implantation strategies.^2,13^ Stress perfusion CMR cohorts show strong agreement with invasive physiology for lesion significance and predictability of outcomes under medical therapy, while viability and infarct characterization refine decisions after myocardial infarction, including the intensity of anti-remodelling regimens and referral to electrophysiology.^15–17^ Population-based series in valve clinics demonstrate improved reproducibility of regurgitant quantification and superior detection of right ventricular dysfunction, strengthening the case for timely intervention and postoperative recovery.^18^ These data have been codified across the ESC corpus and triangulated against a curated PubMed dataset developed for this review to promote transparency and reproducibility.

### CMR in NICE guidelines: a conservative paradigm

NICE develops guidance through appraisal and guideline committees that integrate clinical effectiveness with cost-utility modelling, explicit budget impact thresholds, and deliverability within the NHS.^26^ Within this structure, CMR is endorsed in chronic and acute heart failure when echocardiography is inadequate or diagnostic uncertainty persists.^25,26^ It is positioned after CT first strategies for chest pain and acute coronary syndromes,^27,28^ and considered in valve disease when quantification is uncertain.^29^ There is no cardiomyopathy-specific imaging directive within NICE guidance. Commissioning priorities emphasize echocardiography and CT as default first-line investigations, with CMR utilization shaped by local scanner availability, reporting workforce capacity, and regional contracting frameworks, leading to marked variability in access across the UK. In contrast to the ESC’s evidence-graded integration of emerging modalities, NICE’s appraisal pathway embeds health-economic evaluation and service deliverability, often deferring wider adoption of resource-intensive imaging until both cost-effectiveness and implementation feasibility are demonstrable within NHS commissioning models.

### Comparative disease domain analysis

#### Heart failure

ESC places CMR early in heart failure, Class I for morphology when echocardiography is inadequate, and recommended for tissue characterization to define aetiology and refine risk, informing immunosuppression, genetic testing, and device strategy.^2^ NICE endorses CMR when echocardiography is non-diagnostic but gives limited disease-specific direction, favouring selective use over routine phenotyping.^25,26^ Consequently, ESC pathways deliver earlier mechanistic diagnosis, whereas NICE deploys CMR later to solve residual uncertainty in routine care^2,25,26^ (*Table 3*).

#### Cardiomyopathies

ESC cardiomyopathy guidance makes CMR the principal phenotyping tool, Class I for diagnosis and substrate definition, using late gadolinium enhancement and mapping to distinguish hypertrophic, dilated, arrhythmogenic, inflammatory, and infiltrative disease, and to refine sudden death and heart failure risk.^13,20^ Tissue patterns guide therapy, genetic screening, and defibrillator decisions.^20^ NICE provides no cardiomyopathy-specific imaging standard, creating a gap that defers to local capacity, with variation in access, timing, and equity nationally (*Table 3*). In contrast, the NICE guidance in scope does not provide an explicit imaging sequence when abnormal or non-diagnostic ECG findings (often the initial trigger in cardiomyopathies, myocarditis, and arrhythmic risk evaluation) prompt downstream testing, leaving CMR triage largely to local protocols.

Outcome data underpinning this positioning include meta-analytic evidence that late gadolinium enhancement on CMR in non-ischaemic dilated cardiomyopathy is associated with a higher risk of ventricular arrhythmic events, heart failure hospitalization, and mortality.^30^ In hypertrophic cardiomyopathy, quantitative LGE burden provides incremental risk information for sudden cardiac death beyond conventional clinical markers.^31^

#### Myocarditis and pericarditis

ESC myocarditis/pericarditis guidance positions CMR as a first-line diagnostic tool by integrating oedema and injury markers (including mapping), consistent with the updated Lake Louise criteria.^32^ Prognostically, late gadolinium enhancement in acute myocarditis is associated with increased risk of subsequent adverse cardiac events in meta-analyses.^33^ NICE guidance currently provides limited condition-specific direction for myocarditis imaging, which may reflect topic scope and implementation considerations (including scanner capacity and rapid access pathways) rather than contradictory clinical-effectiveness evidence.

#### Ischaemic heart disease (chronic and acute)

ESC chronic coronary syndromes guidance elevates stress perfusion CMR for diagnosing flow-limiting disease and stratifying risk, with viability to select revascularization.^15^ In NSTE-ACS and STEMI, CMR informs infarct size, microvascular obstruction, and viability, shaping prognosis and treatment.^16,17^ NICE prioritizes CT first for chest pain and reserves CMR for equivocal cases after echocardiography, creating gateway tests, costs, and radiation patterns.^27^ These contrasts influence revascularization yield and the timing of angiography^15^ (*Table 3*). Within NICE chest pain pathways, stress perfusion CMR is typically listed among functional tests when computed tomography coronary angiography (CTCA) is inconclusive or when further functional assessment is required.^34,35^

#### Valvular heart disease

ESC and EACTS position CMR as definitive when echocardiographic data are inadequate or discordant, particularly for quantifying mitral and aortic regurgitation, right ventricular size and function, and aortic dimensions; the recommendation is Class I with Level B evidence.^18^ NICE advises considering CMR when echocardiography is inadequate, without detailed thresholds, leaving sequencing to local teams.^29^ Consequently, ESC pathways may trigger earlier referral for intervention when CMR reveals higher regurgitant burden or adverse remodelling^18,29^ (*Table 3*).

### Discrepancies and clinical consequences

Divergence arises less from contradictory evidence than from timing and positioning. ESC integrates CMR earlier, once analytic validity and clinical utility are considered sufficient, whereas NICE more often adopts selective use after first-line echocardiography or CT triage, leading to different gateways into angiography, electrophysiology, and genetic services.^2,15^ However, direct comparative evidence linking these pathway differences to diagnostic yield, time to diagnosis, or clinical outcomes remains limited. Accordingly, ESC-aligned pathways may facilitate earlier aetiological definition and risk stratification in some contexts, whereas NICE-aligned pathways may defer definitive phenotyping, particularly for myocarditis and cardiomyopathies. Capacity-constrained commissioning may further widen regional access gaps, with potential consequences for treatment allocation, surveillance intensity, and equity across the UK (*Table 3*). Illustrative diagnostic sequencing is summarized in *Graphical abstract*.

In a UK retrospective comparison of two neighbouring NHS hospitals primarily following ESC- vs. NICE-aligned chest pain strategies (*n* = 1321), progression to invasive coronary angiography was similar (9.9 vs. 12.0%) with comparable revascularization rates (4.0 vs. 5.0%), but average investigation costs were lower in the ESC-aligned pathway (£279.66 vs. £325.77; −£46.11 per patient), with UK tariff inputs illustrating the relative contribution of stress echocardiography, CT coronary angiography, and invasive coronary angiography (£176, £220, and £1001, respectively).^4^

### Underlying drivers of divergence

Different evidential thresholds and cost-effectiveness formalisms shape recommendations; NICE’s budget impact orientation and GRADE-to-economic synthesis can down-prioritize early adoption, whereas ESC privileges clinical utility once analytic validity is persuasive.^11,12^ Infrastructure, workforce, commissioning constraints, and conservatism vs. innovation widen the gap between evidence emergence and NHS delivery.^11,12^

Where economic evidence is available, UK analyses suggest that some CMR-based strategies can be cost-effective within conventional NICE opportunity-cost thresholds, although this literature is largely model-based, pathway-specific, and not uniformly generalizable.^36^ Examples include CE-MARC-based modelling supporting CMR in suspected coronary heart disease,^37^ UK modelling of imaging strategies for stable chest pain comparing CMR-guided care against NICE pathways,^38^ and NHS-perspective decision models in acute pathways where CMR can add value after primary percutaneous coronary intervention (PPCI).^39^ For viability assessment, UK health technology assessment modelling has also evaluated contrast-enhanced CMR within competing strategies.^40^ These findings do not remove the practical constraints that NICE must consider; rather, they support interpreting divergence as a product of different institutional mandates, evidence-to-implementation thresholds, and service deliverability. In particular, a conservative sequencing approach may be appropriate in low pre-test probability populations, where incremental benefit is marginal, or where local scanner or reporting capacity constrains timely access and may offset gains.

Costs relevant to CMR implementation include capital and maintenance, scanner time, gadolinium contrast and consumables, and—often most limiting—specialist acquisition and reporting workforce. However, these costs can be partially offset when CMR functions as an effective gatekeeper (for example, by reducing avoidable invasive coronary angiography or serial testing) and when a single multiparametric examination replaces multiple sequential tests. In UK chest pain pathway analyses, ESC-aligned functional strategies and CMR-guided care have shown similar rates of invasive angiography and revascularization with lower or comparable investigation costs relative to NICE-guideline care, supporting interpretation of NICE positioning as driven largely by deliverability and pathway design rather than uniformly negative health-economic signals.^4,38^ Importantly, CT-first pathways also carry material cost, radiation exposure, and capacity implications (scanner time, contrast use, rate-control preparation, high-volume reporting, and potential downstream testing); hence, the relevant question is often optimal sequencing rather than ‘cheap vs. expensive’ modalities. Equally, earlier CMR may not improve the overall efficiency when pre-test probability is low, when CT already answers the dominant clinical question, or when scanner and reporting delays negate the benefit of earlier phenotyping.

These judgements are evidence-informed but remain interpretive because direct comparative trials of guideline-aligned imaging sequences are sparse. Where ESC is likely right: earlier CMR is most defensible in higher-yield phenotypes in which tissue characterization and substrate definition are likely to alter downstream management—particularly cardiomyopathies, suspected myocarditis, and arrhythmic risk contexts where scar pattern or burden may change surveillance, family screening, or device decisions (see Cardiomyopathies and Myocarditis sections). In such settings, delayed access can prolong diagnostic uncertainty and may encourage serial testing. In patients with suspected coronary artery disease, functional CMR also provides ischaemia testing alongside assessment of ventricular function and myocardial scar or fibrosis. Emerging haemodynamic mapping approaches may further expand this role, although current clinical experience remains limited.^41,42^

Where NICE may be justified: a CT-first strategy is rational in lower pre-test probability chest pain populations, where exclusion of obstructive coronary disease is often the dominant initial question. CTCA may also identify non-obstructive atherosclerosis or coronary remodelling that can inform preventive strategies. However, any CT-first strategy remains dependent on local scanner access, reporting capacity, and the need for downstream functional testing in equivocal cases.

Overall, binary ‘CT-first’ or ‘CMR-for-all’ positioning may both be suboptimal. A hybrid approach—CTCA first in low-risk stable chest pain, but selective early CMR in patients with abnormal ECG or troponin patterns, suspected cardiomyopathy or myocarditis, discordant echocardiography, or a specific need for functional assessment—may better align evidential value with capacity. This proposition remains inferential rather than trial-proven, but it may offer a pragmatic route for publicly funded systems facing simultaneous pressures of evidence, cost containment, and deliverability.

### Limitations

This review synthesized formal ESC and NICE guidance rather than individual-patient or trial-level data; therefore, conclusions reflect the interpretation of each organization’s published processes rather than independent evidence generation. Differences in scope, update cycles, and evidence cut-off dates may contribute to apparent discrepancies in CMR positioning. Although guideline selection and extraction were conducted systematically using a transparent framework, class- and level-grading systems are not directly interchangeable, and mapping across frameworks introduces some interpretative subjectivity. Economic and commissioning inferences were derived from publicly available documents and may not fully capture regional implementation nuances or unpublished cost-effectiveness analyses. Finally, the synthesis focused on major adult cardiovascular conditions with mature imaging pathways; findings may not generalize to congenital, paediatric, or rare disease contexts.

#### Opportunities for alignment

The UK remains at the forefront of CMR adoption, supported by world-leading registries, specialist centres, and robust training infrastructure. NICE’s more selective positioning of CMR can be framed as contextually constrained by differing institutional mandates (including explicit cost-utility and implementability requirements) rather than as an intrinsic deficit. Alignment efforts should therefore focus on shared outcomes and local feasibility, informed by capacity and commissioning realities.

## Conclusion

ESC places CMR early with graded, disease-specific weight, whereas NICE adopts conservatively around cost and service constraints. Harmonized, evidence-linked pathways, shared outcome registries, and targeted commissioning could unify imaging guidance, protecting equity while preserving fiscal responsibility and practical implementation.

## Supplementary Material

qyag079_Supplementary_Data

## Data Availability

All data are incorporated into the article and its online Supplementary material.

## Lead author biography

Dr Pankaj Garg is an Associate Professor in Cardiovascular Medicine at the University of East Anglia, Norwich, UK. and Honorary Consultant Cardiologist at Norfolk and Norwich University Hospitals. He graduated in Medicine with Honours; he became a member of the Royal College of Physicians in 2007. After completing his BHF-funded PhD at Leeds (2017), he was awarded NIHR Clinical Lectureship programme at the University of Sheffield. In 2020, he was awarded Wellcome Trust Clinical Research Career Development Fellowship. His research focus is cardiovascular haemodynamics and Cardiac Magnetic Resonance Imaging. His ambition is to discover and advance a better understanding of the interface between cardiovascular flow and cardiac remodelling. He has received 20 national/international awards and has published >200 peer-reviewed papers.
