# Supplementary material for: Cardiovascular magnetic resonance in contemporary guidelines: divergence between ESC and NICE across major cardiovascular diseases
Source: Eur Heart J Imaging Methods Pract. 2026 Apr 22;4(1):qyag079. doi: 10.1093/ehjimp/qyag079 (PMC13180753; doi:10.1093/ehjimp/qyag079)
Supplement: qyag079_Supplementary_Data [file qyag079_supplementary_data.docx]

# Supplementary Tables: Appraisal Framework Application and Guideline Quality Assessment

## Supplementary Table S1. How PRISMA, AGREE II, GRADE and CHEERS were applied in this review

| **Tool** | **Primary purpose** | **Unit of assessment** | **Approach in this manuscript** | **Outputs** |
| --- | --- | --- | --- | --- |
| PRISMA 2020 | Reporting framework for transparent search/screening and study selection | Search and selection process | Used to structure reporting of identification, screening, eligibility and inclusion; adapted for guideline corpus rather than trial meta-analysis. | Flow diagram (Fig.1) and explicit description of search/screening in Methods. |
| AGREE II | Appraisal of guideline development quality and reporting | Included guidance documents (ESC and NICE) | Applied qualitatively at domain level (Scope & purpose; Stakeholder involvement; Rigour; Clarity; Applicability; Editorial independence); no item-by-item scoring or inter-rater reliability. | Domain-level strengths/limitations summarised per included document (Supplementary Table S2). |
| GRADE (concepts) | Interpretive framework for certainty and recommendation strength | Evidence statements underpinning recommendations | Used as an interpretive lens (limitations/risk of bias, inconsistency, indirectness, imprecision, publication bias) to explain how similar evidence bases can map differently to ESC class/level versus NICE directives; no de novo re-grading of evidence profiles. | Narrative explanation in Methods/Results/Discussion plus mapping rules and limitations (Supplementary Table S3). |
| CHEERS 2022 | Reporting standards for health economic evaluations | Economic considerations referenced within NICE guidance and supporting literature | Used as a conceptual benchmark to structure extraction (perspective, comparators, time horizon, outcomes, costs, uncertainty, thresholds) rather than as a checklist-based critical appraisal of each model. | Structured reporting of economic/commissioning drivers in Discussion plus extracted economic evidence fields (Supplementary Table S4). |

## Supplementary Table S2. Qualitative AGREE II domain appraisal of included ESC and NICE guidance documents

| Guidelines (Year) | Guidance document (short title) | Scope & purpose | Stakeholder involvement | Rigor of development | Clarity of presentation | Applicability | Editorial independence (incl. COI reporting) |
| --- | --- | --- | --- | --- | --- | --- | --- |
| ESC (2023) [1] | Cardiomyopathies guideline | High | Moderate–High | High | High | Moderate | High (COI disclosed; industry relationships managed variably across task forces) |
| ESC (2021) [2] | Heart failure guideline | High | Moderate–High | High | High | Moderate | High (COI disclosed; industry relationships managed variably across task forces) |
| ESC (2025) [3] | Myocarditis & pericarditis guideline | High | Moderate–High | High | High | Moderate | High (COI disclosed; industry relationships managed variably across task forces) |
| ESC (2019) [4] | Chronic coronary syndromes guideline | High | Moderate–High | High | High | Moderate | High (COI disclosed; industry relationships managed variably across task forces) |
| ESC (2020) [5] | NSTE-ACS guideline | High | Moderate–High | High | High | Moderate | High (COI disclosed; industry relationships managed variably across task forces) |
| ESC (2017) [6] | STEMI guideline | High | Moderate–High | High | High | Moderate | High (COI disclosed; industry relationships managed variably across task forces) |
| ESC (2021) [7] | Valvular heart disease guideline | High | Moderate–High | High | High | Moderate | High (COI disclosed; industry relationships managed variably across task forces) |
| ESC (2020) [8] | Adult congenital heart disease guideline | High | Moderate–High | High | High | Moderate | High (COI disclosed; industry relationships managed variably across task forces) |
| ESC (2022) [9] | Ventricular arrhythmias & SCD guideline | High | Moderate–High | High | High | Moderate | High (COI disclosed; industry relationships managed variably across task forces) |
| ESC (2020) [10] | Atrial fibrillation guideline | High | Moderate–High | High | High | Moderate | High (COI disclosed; industry relationships managed variably across task forces) |
| ESC (2015) [11] | Pericardial diseases guideline | High | Moderate–High | High | High | Moderate | High (COI disclosed; industry relationships managed variably across task forces) |
| ESC (2022) [12] | Pulmonary hypertension guideline | High | Moderate–High | High | High | Moderate | High (COI disclosed; industry relationships managed variably across task forces) |
| ESC (2023) [13] | Infective endocarditis guideline | High | Moderate–High | High | High | Moderate | High (COI disclosed; industry relationships managed variably across task forces) |
| NICE (2018) [14] | Chronic heart failure (NG106) | High | High | High | High | High | High (committee process; COI policies and declarations provided) |
| NICE (2014) [15] | Acute heart failure (CG187) | High | High | High | High | High | High (committee process; COI policies and declarations provided) |
| NICE (2010) [16] | Chest pain of recent onset (CG95) | High | High | High | High | High | High (committee process; COI policies and declarations provided) |
| NICE (2020) [17] | Acute coronary syndromes (NG185) | High | High | High | High | High | High (committee process; COI policies and declarations provided) |
| NICE (2021) [18] | Heart valve disease (NG208) | High | High | High | High | High | High (committee process; COI policies and declarations provided) |

Notes: Ratings are qualitative (High/Moderate/Variable) to summarise domain-level strengths/limitations using AGREE II constructs; they are not item-level AGREE II scores and do not represent inter-rater results.

## Supplementary Table S3. Interpretive mapping of ESC and NICE recommendation formats using GRADE concepts

| **Element** | **ESC format** | **NICE format** | **How mapped/used in this review (interpretive)** |
| --- | --- | --- | --- |
| Strength of recommendation | Class I/IIa/IIb/III | Directive language (e.g., offer/consider/do not offer) with pathway sequencing | Strength inferred from class and wording; comparisons focus on sequencing and thresholds rather than forcing numeric equivalence. |
| Certainty/quality of evidence | Level A/B/C | Evidence statements and evidence reviews underpin guideline recommendations; explicit certainty grading varies by guideline type | GRADE domains used to discuss why certainty may be judged differently (indirectness, applicability to NHS, heterogeneity, etc.); no re-grading performed. |
| Upgrading/downgrading drivers | Trial/registry evidence; generalisability across Europe; clinical utility once analytic validity is established | Cost-utility and budget impact; implementation feasibility; topic scope; opportunity cost considerations | Mapped narratively to explain divergences without implying that one system is ‘wrong’; emphasises mandate and constraints. |

## Supplementary Table S4. CHEERS-informed economic evidence extraction fields (examples relevant to UK practice)

| Articles | Clinical context | Perspective/setting | Comparator(s) | Outcome metric(s) | Key CHEERS fields captured in extraction (examples) |
| --- | --- | --- | --- | --- | --- |
| Walker et al., 2013 [19] | Suspected CHD (CE-MARC-based strategies) | UK/NHS modelling | CMR vs SPECT and other strategies | Cost per QALY; diagnostic pathway costs | Perspective; comparators; model structure; costs; outcomes; uncertainty |
| Walker et al., 2021 [20] | Stable chest pain / suspected CAD | UK setting (Heart/BMJ publication) | CMR-guided care vs MPS and NICE-guideline care | QALYs; costs; probability of cost-effectiveness | Time horizon; costs/outcomes; sensitivity analyses; decision uncertainty |
| Stokes et al., 2019 [21] | PPCI pathway activation (acute settings) | UK/NHS | Usual care with vs without CMR | Costs; outcomes (modelled); QALYs | Perspective; resource use; assumptions; uncertainty |
| Campbell et al., 2014 [22] | Myocardial viability in IHD | UK HTA modelling | CE-CMR vs PET/other strategies | Cost per QALY; EVPI | Threshold analysis; probabilistic sensitivity analysis; uncertainty reporting |

Notes: CHEERS was used to structure what information was extracted and reported. This supplementary material does not provide a full CHEERS checklist score for each study unless explicitly required in future revisions.

**Reference**

1. Arbelo E, Protonotarios A, Gimeno JR, Arbustini E, Barriales-Villa R, Basso C, et al. 2023 ESC Guidelines for the management of cardiomyopathies. Eur Heart J. 2023 Oct 1;44(37):3503–626.

2. McDonagh TA, Metra M, Adamo M, Gardner RS, Baumbach A, Böhm M, et al. 2021 ESC Guidelines for the diagnosis and treatment of acute and chronic heart failure. Eur Heart J. 2021 Sep 21;42(36):3599–726.

3. Schulz-Menger J, Collini V, Gröschel J, Adler Y, Brucato A, Christian V, et al. 2025 ESC Guidelines for the management of myocarditis and pericarditis. Eur Heart J. 2025 Oct 22;46(40):3952–4041.

4. Knuuti J, Wijns W, Saraste A, Capodanno D, Barbato E, Funck-Brentano C, et al. 2019 ESC Guidelines for the diagnosis and management of chronic coronary syndromes. Eur Heart J. 2020 Jan 14;41(3):407–77.

5. Collet JP, Thiele H, Barbato E, Barthélémy O, Bauersachs J, Bhatt DL, et al. 2020 ESC Guidelines for the management of acute coronary syndromes in patients presenting without persistent ST-segment elevation. Eur Heart J. 2021 Apr 7;42(14):1289–367.

6. Ibanez B, James S, Agewall S, Antunes MJ, Bucciarelli-Ducci C, Bueno H, et al. 2017 ESC Guidelines for the management of acute myocardial infarction in patients presenting with ST-segment elevation: The Task Force for the management of acute myocardial infarction in patients presenting with ST-segment elevation of the European Society of Cardiology (ESC). Eur Heart J. 2018 Jan 7;39(2):119–77.

7. Vahanian A, Beyersdorf F, Praz F, Milojevic M, Baldus S, Bauersachs J, et al. 2021 ESC/EACTS Guidelines for the management of valvular heart disease: Developed by the Task Force for the management of valvular heart disease of the European Society of Cardiology (ESC) and the European Association for Cardio-Thoracic Surgery (EACTS). European Heart Journal. 2022 Feb 14;43(7):561–632.

8. Baumgartner H, De Backer J, Babu-Narayan SV, Budts W, Chessa M, Diller GP, et al. 2020 ESC Guidelines for the management of adult congenital heart disease. Eur Heart J. 2021 Feb 11;42(6):563–645.

9. Zeppenfeld K, Tfelt-Hansen J, de Riva M, Winkel BG, Behr ER, Blom NA, et al. 2022 ESC Guidelines for the management of patients with ventricular arrhythmias and the prevention of sudden cardiac death. Eur Heart J. 2022 Oct 21;43(40):3997–4126.

10. Hindricks G, Potpara T, Dagres N, Arbelo E, Bax JJ, Blomström-Lundqvist C, et al. 2020 ESC Guidelines for the diagnosis and management of atrial fibrillation developed in collaboration with the European Association for Cardio-Thoracic Surgery (EACTS): The Task Force for the diagnosis and management of atrial fibrillation of the European Society of Cardiology (ESC) Developed with the special contribution of the European Heart Rhythm Association (EHRA) of the ESC. Eur Heart J. 2021 Feb 1;42(5):373–498.

11. Adler Y, Charron P, Imazio M, Badano L, Barón-Esquivias G, Bogaert J, et al. 2015 ESC Guidelines for the diagnosis and management of pericardial diseases: The Task Force for the Diagnosis and Management of Pericardial Diseases of the European Society of Cardiology (ESC)Endorsed by: The European Association for Cardio-Thoracic Surgery (EACTS). Eur Heart J. 2015 Nov 7;36(42):2921–64.

12. Humbert M, Kovacs G, Hoeper MM, Badagliacca R, Berger RMF, Brida M, et al. 2022 ESC/ERS Guidelines for the diagnosis and treatment of pulmonary hypertension. Eur Heart J. 2022 Oct 11;43(38):3618–731.

13. Delgado V, Ajmone Marsan N, de Waha S, Bonaros N, Brida M, Burri H, et al. 2023 ESC Guidelines for the management of endocarditis. Eur Heart J. 2023 Oct 14;44(39):3948–4042.

14. National Guideline Centre (UK). Chronic Heart Failure in Adults: Diagnosis and Management [Internet]. London: National Institute for Health and Care Excellence (NICE); 2018 [cited 2026 Jan 22]. (National Institute for Health and Care Excellence: Guidelines). Available from: http://www.ncbi.nlm.nih.gov/books/NBK536075/

15. National Clinical Guideline Centre (UK). Acute Heart Failure: Diagnosing and Managing Acute Heart Failure in Adults [Internet]. London: National Institute for Health and Care Excellence (UK); 2014 [cited 2026 Jan 22]. (National Institute for Health and Care Excellence: Clinical Guidelines). Available from: http://www.ncbi.nlm.nih.gov/books/NBK248063/

16. Recent-onset chest pain of suspected cardiac origin: assessment and diagnosis [Internet]. London: National Institute for Health and Care Excellence (NICE); 2016 [cited 2026 Feb 8]. (National Institute for Health and Care Excellence: Guidelines). Available from: http://www.ncbi.nlm.nih.gov/books/NBK553650/

17. Acute coronary syndromes [Internet]. London: National Institute for Health and Care Excellence (NICE); 2020 [cited 2026 Feb 8]. (National Institute for Health and Care Excellence: Clinical Guidelines). Available from: http://www.ncbi.nlm.nih.gov/books/NBK565352/

18. Heart valve disease presenting in adults: investigation and management [Internet]. London: National Institute for Health and Care Excellence (NICE); 2021 [cited 2026 Feb 8]. (National Institute for Health and Care Excellence: Guidelines). Available from: http://www.ncbi.nlm.nih.gov/books/NBK577831/

19. Walker S, Girardin F, McKenna C, Ball SG, Nixon J, Plein S, et al. Cost-effectiveness of cardiovascular magnetic resonance in the diagnosis of coronary heart disease: an economic evaluation using data from the CE-MARC study. Heart. 2013 Jun;99(12):873–81.

20. Walker S, Cox E, Rothwell B, Berry C, McCann GP, Bucciarelli-Ducci C, et al. Cost-effectiveness of cardiovascular imaging for stable coronary heart disease. Heart. 2021 Mar;107(5):381–8.

21. Stokes EA, Doble B, Pufulete M, Reeves BC, Bucciarelli-Ducci C, Dorman S, et al. Cardiovascular magnetic resonance in emergency patients with multivessel disease or unobstructed coronary arteries: a cost-effectiveness analysis in the UK. BMJ Open. 2019 Jul 11;9(7):e025700.

22. Campbell F, Thokala P, Uttley LC, Sutton A, Sutton AJ, Al-Mohammad A, et al. Systematic review and modelling of the cost-effectiveness of cardiac magnetic resonance imaging compared with current existing testing pathways in ischaemic cardiomyopathy. Health Technol Assess. 2014 Sep;18(59):1–120.
